# Pull-Up Palms (PUP): A Case of Exercise-Associated Palmar Callosities and Review of Sports-Related Hand-Associated Skin Conditions in Athletes

**DOI:** 10.7759/cureus.34476

**Published:** 2023-01-31

**Authors:** David W Hoyt, Philip R Cohen

**Affiliations:** 1 School of Medicine, Spencer Fox Eccles School of Medicine at University of Utah, Salt Lake City, USA; 2 Dermatology, University of California, Davis Medical Center, Sacramento, USA

**Keywords:** athletes, calluses, callosities, dermatosis, exercise, hand, palms, pull-up, skin, sports

## Abstract

Sports dermatology describes skin conditions occurring in athletes. We describe a man with callosities on his palmar hands and fingers secondary to pull-ups and review sports-related dermatoses involving the hands. A 42-year-old man presented with a several-year history of calluses on his palmar hands. The lesions correspond to areas of contact on his ventral hand with the pull-up bar; therefore, the condition is referred to as pull-up palms (PUP). Sports-related dermatoses affecting the hands include contact dermatitis, infections, lacerations, and mechanical trauma. Several of the sports-associated conditions of the hand are unique to a specific sport. Hand-associated sports dermatoses are reviewed.

## Introduction

Sports dermatology refers to acquired skin disorders in athletes. They include not only infections, but also dermatoses from trauma and contact allergies. Several sports have unique skin conditions that may be observed in participants [1-20].

The hands are used in many sports, both individual and team. They include sports with balls, racquets, or both. In addition, they include athletic activities requiring firm gripping of mobile or stationary bars such as gymnastics and weight lifting [1-20].

A man presented with callosities on his palmar distal hand and proximal digits; he was an avid exerciser and did repetitive pull-ups each day. His lesions were similar in location and morphology to those observed in weight lifters. We suggest that these skin changes be referred to as pull-up palms. We also review some of the sports-related skin conditions of the hands in athletes.

## Case presentation

A 42-year-old healthy man presented for a total body skin evaluation. He was taking no medications and had no allergies. A dysplastic nevus on his right deltoid area had been removed three years previous; several other benign pigmented lesions had also previously been biopsied.

**Figure 1:**
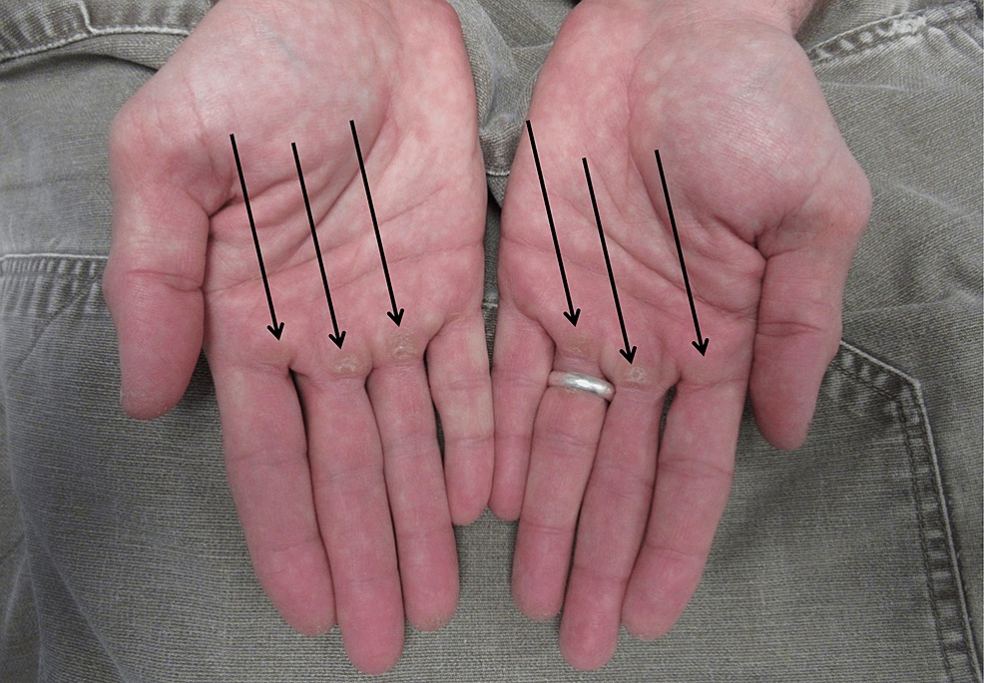
Pull-up palms (PUP) presenting as ventral hand and finger callosities Distant view of palmar callosities (black arrows) on the hands of a 42-year-old man who does pull-ups for more than 30 minutes each day during the previous five years.

**Figure 2:**
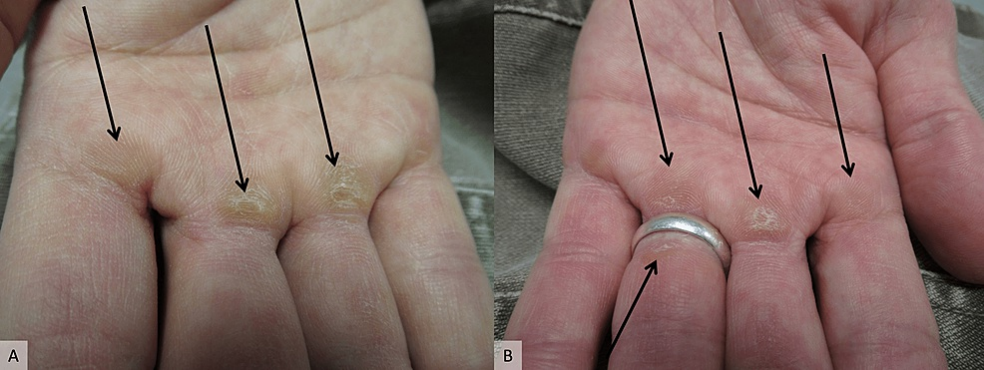
Palmar callosities are the morphologic presentation of exercise-associated pull-up palms (PUP) Closer views of the right hand (A) and left hand (B) show callosities (black arrows) on the palmar distal hand and proximal second, third, and fourth digits on the right and left hands in an exercise enthusiast who does daily pull-ups. A notable asymmetry between hands is a small callosity just distal to the patient’s wedding band on his left fourth digit.

Cutaneous examination did not show any concerning pigmented lesions. However, evaluation of his palms showed callosities located bilaterally on the distal ventral hands and proximal second, third, and fourth digits (Figures 1, 2). The patient reported that he did a minimum of 50 pull-ups, using both underhand and overhand grip, each day for the past five years.

Correlation of his clinical history and palmar lesion morphology established a diagnosis of palmar callosities; based on his exercise-related activity and the location of the lesions, we have referred to his sports-associated condition as pull-up palms. The patient considered his palmar callosities to be a testament to his physical fitness and did not desire any treatment. No other sports-related dermatologic conditions were observed.

## Discussion

Sports dermatology includes cutaneous conditions resulting from not only non-aquatic activities but also sports in either fresh water or salt water. The dermatologic disorders that may develop in athletes can be a direct consequence of the actions involved by the participant or an indirect sequelae of infection or allergy. Similar to our patient with pull-up palms, the designation of specific sports-associated dermatosis has been created by linking the athletic activity and the affected location [1-20].

The hands can be affected by several sports-associated dermatoses (Table 1) [1-20]. These include infections from bacteria, fungi, and viruses. They also include contact dermatitis, lacerations, mechanical trauma, and miscellaneous conditions [1-20].

**Table 1:** Hand-associated sports dermatoses Abbreviations: CR, current report; POTASH, post ambulatory swollen hands; PUP, pull-up palms ^a^Bicycle cuff-associated thiuram allergic contact dermatitis. ^b^Allergic contact dermatitis to epoxy resin. ^c^Plant-associated allergic contact dermatitis to lichen in a rock climber. ^d^Also seen in individuals who play marbles. ^e^Pull-up palms can also be referred to as localized acquired keratoderma associated with pull-ups. ^f^Similar palmar changes have also been observed in gymnasts, rowers, and participants of racquet-held sports such as tennis. ^g^Development of aquagenic syringeal acrokeratoderma in a coxswain.

Condition	Reference
Bacterial infection	[1,2]
Impetigo	[1]
Pitted keratolysis	[2]
Contact dermatitis	[3-7]
Bicycle cuff dermatitis^a^	[3]
Bowler’s grip	[4]
Golf club dermatitis^b^	[5]
Rock climber dermatitis^c^	[6]
Trekking blisters	[7]
Fungal infections	[8-10]
Erosio interdigitalis blastomycetica	[8]
Onychomycosis	[9]
Tinea manuum	[10]
Lacerations	[11,12]
Karate cicatrices	[11]
Rugby wounds	[12]
Mechanical trauma	[13-17]
Athlete’s nodules^d^	[13]
Boxer’s knuckle pads	[14]
Canyoning hands	[15]
Pool palms	[16]
Pull-up palms (PUP)^e,f^	CR
Weight lifter’s calluses^f^	[14,17]
Miscellaneous	[18,19]
Post ambulatory swollen hands (POTASH)	[18]
Watersport hands^g^	[19]
Viral infections	[20]
Boxing glove herpes	[20]

High temperature, moisture, and traumatic damage to the skin make athletes more susceptible to cutaneous bacterial infections. *Staphylococcus aureus* and *Streptococcus pyogenes* are the predominant causal microbes of impetigo, which can involve the hands. Management of bacterial infections of the hands includes oral or topical antibiotics and appropriate wound care [1].

Corynebacterial infections are another common etiology of bacterial infection among athletes, primarily causing pitted keratolysis. Other bacteria have also been implicated in the pathogenesis of pitted keratolysis: *Actinomyces* species, *Dermatophilus* species, *Micrococcus sedentarius* (which has been renamed as *Kytococcus sedentarius*), and *Streptomyces* species. Though pitted keratolysis primarily affects the feet, one study reported 18 individuals with pitted keratolysis of the hands [2].

Fungal infections are common among athletes; however, they more frequently involve the feet than the hands. Erosio interdigitalis blastomycetica refers to infection from *Candida albicans* in the web space between the third and fourth digits. Erosio interdigitalis blastomycetica requires moist conditions to develop; therefore, it should be considered in athletes who often wear gloves, are frequently in water, or experience excessive sweating. Management should include avoiding occlusion of the affected site, keeping hands dry, and topical or oral antifungal agents in resistant cases [8].

*Trichophyton tonsurans *has caused epidemic tinea outbreaks among contact sports teams. A high school judo athlete with tinea manuum presented with *T. tonsurans* culture-positive onychomycosis of the left middle fingernail. Over one-third of his teammates were also infected with *T. tonsurans* [9].

Another study reported that 11.5% of combat sport gyms in Japan were positive for *T. tonsurans*. The investigators documented one case of tinea manuum. Oral and topical antifungal drugs have been shown to be effective in treating fungal infections of the hands [10].

Viral infections of the skin, especially herpes simplex virus type 1, are most often found on the head, neck, and trunk in athletes. A 35-year-old man presented with crusted erythematous papules and vesicles on his knuckles after using communal boxing gloves at his gym, and reported similar symptoms in three other gym members. Cutaneous herpes simplex virus type 1 infection was diagnosed by serology, cultures, and biopsy. These virus-induced lesions, termed “boxing glove herpes”, resolved spontaneously in all four individuals within three weeks of discontinuing the use of the communal boxing gloves [20].

Many offending allergens are known to cause contact dermatitis. However, sport-specific contact allergens are less frequently described. Allergy testing and exposure reduction are useful in determining etiology and limiting exacerbations of sports-induced contact dermatitis [3-7].

Bicycling requires gripping handlebars for the duration of the exercise. A 28-year-old woman, who rode a bicycle to her university every day, presented with bilateral erythematous and hyperkeratotic eczema on the palmar hands. Allergy testing was positive for thiurams, which are common rubber additives used in bicycle cuffs that cover the handlebars and are gripped by the rider. Her symptoms were resolved by consistently wearing cloth gloves while riding her bicycle [3].

Bowler’s rosin (used to enhance grip) and epoxy resin (used in the manufacture of bowling balls) have been reported as causing allergic contact dermatitis of the hands in bowlers and bowling shop workers. An 84-year-old woman presented with pruritic erythematous scaly patches on the face, neck, back, trunk, and extremities. She bowled frequently, using rosin every time. After a thorough review of potential triggers and positive allergy testing for colophony, she was advised to stop using “bowler’s grip” rosin and treated with topical corticosteroids [4].

Golfers are also at risk of developing contact dermatitis of the hands. A 71-year-old man who golfed regularly and repaired golf clubs developed erythematous scaly dermatitis with vesicles on his bilateral palms and digits. He had been working as a golf club repairman for four years at the time of his presentation. Standard patch testing demonstrated a positive reaction to diglycidyl ether of bisphenol A, a type of epoxy resin used on golf club handles. Discontinuing his work as a golf club repairman and wearing gloves when handling golf clubs resolved his symptoms, except for dry hands [5].

Athletes whose sport takes them to the mountains and forests are at risk of developing palmar contact dermatitis from certain plants. Lichen exposure is uncommon, but was reported as causing an itchy, eczematous rash on the hands of a 34-year-old man who rock climbed outdoors. The man had a history of hay fever, pet allergy, and asthma; he reported developing a pruritic rash over his face, neck, forearms, and hands after rock climbing outdoors; however, he experienced no symptoms after indoor climbing. Patch testing confirmed the diagnosis of lichen dermatitis, and the patient was advised to avoid outdoor rock climbing [6].

Sport equipment-induced contact dermatitis was observed in a 27-year-old woman who developed bilateral hand swelling, with coalescing papules and vesicles over the dorsum of both hands, while trekking in Nepal. She had been using trekking poles with straps around the hands for several days and reported no other temperature or chemical exposures. It was determined that the hand straps on the trekking poles contained the offending allergen, and the patient’s symptoms resolved within two weeks of oral corticosteroids and antihistamines along with topical steroids and antibiotic ointment [7].

Contact sports and athletic activities requiring studded footwear put the participants at higher risk of laceration injury to the hands. Multiple scattered, hypopigmented, round and linear, minimally atrophic patches developed bilaterally on the dorsal hands of a 46-year-old man who participated in karate from age 13 until age 27. They were termed “karate cicatrices”. Most of the lacerations resulted from contact with nails, teeth, and bricks while punching and blocking in karate [11].

Another study reported that skin injuries and lacerations accounted for 5.1% of injuries during professional and amateur male rugby matches. Indeed, these injuries resulted in the rugby player missing time participating during the match due to the injury. While the high speed, contact nature of the sport makes traumatic injury nearly inevitable, laceration risk in both karate and rugby athletes could be decreased by the regular use of hand protection and mouth guards [11,12].

Mechanical dermatoses of the hands, such as pull-up palms, often result from repeated frictional stress to the skin. Indeed, the causative equipment that is used is often unique to the specific sport. In addition to the legs and feet of surfers, collagenous connective tissue nevi (collagenomas) have also been observed on the knuckles of boxers and marble players, likely caused by recurrent trauma and friction. Collagenomas acquired from participation in sports have been referred to as athlete’s nodules. These nodules are often asymptomatic; however, they can be painful and may limit participation in the causative sport. Therapeutic options for athlete’s nodules include keratolytics, topical corticosteroids, pumice stone paring, or surgical excision [13].

In addition to athlete’s nodules, boxers are also at risk for developing benign “knuckle pads”. These nodules occur on the dorsal aspect of the metacarpophalangeal joints or the proximal interphalangeal joint, are often painless, and can be mistaken for arthritis. A 21-year-old man who participated in boxing presented with painful hyperkeratotic, fissured callosities over most of his proximal interphalangeal joints and some metacarpophalangeal joints on both hands. Although he did not participate in top-level competition, he confirmed wearing boxing gloves and practicing boxing five times weekly for the previous six months [14].

Outdoor sports can also present with irritant dermatitis. Individuals who participate in canyoneering (a sport that involves rappelling, scrambling on wet and dry rocks, and swimming down water rapids) often present with “canyoning hand”: erythema and erosions on the fingertips and thenar and hypothenar eminences after participating in canyoning. This dermatitis results from repeated contact between the participant’s wet hands and the abrasive rocks and ropes. Decreased sensation in the hands due to cold water exposure and heightened emotions results in more severe damage before the injury is first noticed by the athlete [15].

Aquatic activities can involve direct contact with high-friction surfaces such as diving boards and non-slip cement that can cause injury to the hand. Friction-induced dermatoses on the ventral fingers and hands following aquatic activities have been referred to as pool palms and are caused by repeated contact with the rough cement on the bottom of the pool. Pool palms are most often described in children; they can range in severity from tender erythematous lesions to painful blisters. Pool palms can be prevented by eliminating exposure to the pool. Symptomatic treatment of pool palms can be provided by soaking the hands in cold water, which may relieve pain during the healing process [16].

Stationary exercise and strength training equipment can also cause friction-induced and pressure-induced hand injury. Our patient developed non-tender, bilateral palmar callosities; he performed pull-ups for an extended duration each day. Weight training-induced palmar keratoderma over the surface of the metacarpophalangeal joints has been termed “weight lifter’s calluses”. In addition to exercises such as pull-ups and lifting weights, sports associated with chronic and repeated frictional trauma to the hands, such as gymnastics, rowing, and racquet sports including tennis, can result in calluses on the palms [14,17].

The calluses created by exercise and sports-related activities usually do not have any functional repercussions or medical implications. Indeed, their treatment depends upon the aesthetic preference of the affected individual. Similar to our patient with pull-up palms, patients with weight lifter’s calluses often refuse treatment because they feel the calluses reflect not only their dedication to the activity, but also their accomplishment in the sport [17].

However, there are management options for those individuals who want to treat their exercise-induced and sports activity-related palmar keratoderma. These include paring with a pumice stone or razor and topical keratolytics. In addition, wearing gloves while exercising may prevent palmar calluses from occurring or progressing [17].

The pathophysiology of some sports-related dermatoses remains to be established. Aquagenic wrinkling of the palms, referred to as aquagenic syringeal acrokeratoderma or “watersport hands”, is characterized as small hypopigmented papules and plaques on the palms and soles after submersion in water. A 19-year-old woman collegiate coxswain presented with three weeks of watersport hands, accompanied by burning, pruritus, and a tightening sensation. Her symptoms worsened with water exposure but were persistent throughout the day. Though the etiology remains unclear, watersport hands demonstrate a predilection for women in their teens and twenties; it is associated with cyclo-oxygenase inhibitor use, cystic fibrosis, and asthma. The lesions may not resolve spontaneously; persistent lesions can be treated with aluminum chloride, botulinum toxin, iontophoresis, and salicylic acid ointment [19].

Although walking and jogging do not directly involve the hands, these activities are associated with a unique dermatosis of the hands. Post ambulatory swollen hands (POTASH) refers to the benign bilateral swelling of the hands following walking, hiking, or running. In one patient’s experience, the degree of hand swelling was proportional to the distance ran, exhibiting a positive fist sign (demonstrated by an inability to make a fist when attempting to clench the fingers) after one hour of running. The exact pathophysiology of post ambulatory hand swelling remains to be established; however, most cases are asymptomatic and resolve spontaneously [18].

## Conclusions

Sports-related dermatoses develop in athletic participants. Some of the dermatologic conditions are associated with a specific activity or a specific body location, or both. A man developed callosities on his palmar hands and fingers secondary to repetitive pull-up exercises; in order to acknowledge the sport and the location of the participant’s dermatosis, we refer to his condition as pull-up palms (PUP). Similar to sports participants and exercise enthusiasts with athlete’s nodules and weight lifter’s callosities, he was proud of his palmar calluses that had developed from his daily exercise and did not desire treatment.
